# Characteristics and phylogenetic analysis of the complete chloroplast genome of *Primulina hedyotidea*

**DOI:** 10.1080/23802359.2023.2238932

**Published:** 2023-09-23

**Authors:** Haicheng Wen, Yongjing Su, Ao Xie, Chen Lin, Wei Wei

**Affiliations:** Guangxi University of Chinese Medicine, Guangxi, P.R. China

**Keywords:** Chloroplast genome, *Primulina hedyotidea*, phylogenetic tree

## Abstract

*Primulina hedyotidea* (Woon Young Chun) Yin Zheng Wang 2011 is an important medicinal plant that has a long history of medicinal use in China. In this experiment, the whole chloroplast genome of *P. hedyotidea* was determined by next-generation sequencing technology. The total base length of *P. hedyotidea* was 153,297 bp, the GC content was 37.62%, the inverted repeat (IR) region length was 25,494 bp, the large single copy (LSC) region was 84,158 bp and the small single copy (SSC) region was 18,151 bp. In addition, the genome consisted of 80 protein-coding genes, 4 rRNA genes, and 28 tRNA genes, for a total of 112 genes. A phylogenetic tree was constructed to explore the evolutionary relationship between *P. hedyotidea* and other species. The findings of phylogenetic tree analysis show that *Primulina huaijiensis* and *P. hedyotidea* have a close relationship, and this study can help with species identification and phylogenetic analysis within *Primulina* and Gesneriaceae species.

## 1. Introduction

*Primulina hedyotidea* (Woon Young Chun) Yin Zheng Wang 2011 is a plant species of Gesneriaceae. It is widely distributed in tropical regions of China. In Chinese folk medicine, it is commonly used to treat conditions such as eczema, urticaria, psoriasis, bone fractures, and trauma. *Primulina hedyotidea* is the same as *Didymocarpus hedyotideus*. Its main chemical components are 6-hydroxy-alpha-dunnione (1), methyl 1,1′,4,4′-tetrahydro-3-hydroxy-1,1′,4,4′-tetraoxo[2,2′-binaphthalene]-3′-carboxylate (2), didymocarpuslignan A (3), didymocarpuslignan B (4), didymocarpuslignan C (5), and 7 R,7′’R,8S,8′’S-(+) icariol A2 (6), as well as additional steroids, lignans, anthraquinones, and naphthoquinones; while evaluating the anti-hepatitis B virus activities of compounds 3–6, compounds 3–5 demonstrated *in vitro* antiviral effects on the hepatitis B virus (Xiao et al. 2011; Tang et al. 2014). Although there are existing studies on the chemical constituents of *P. hedyotidea*, its pharmacological actions and complete chloroplast (cp) genome characteristics remain unexplored. Thus, studying and analyzing the complete chloroplast genome of *P. hedyotidea* will help with species identification, clinical safe drug use, and resource protection for *P. hedyotidea*. It will also explain the plant’s phylogenetic position in the family Gesneriaceae and its genetic relationship with other *Primulina* plants.

## 2. Materials and methods

**Figure 1.**
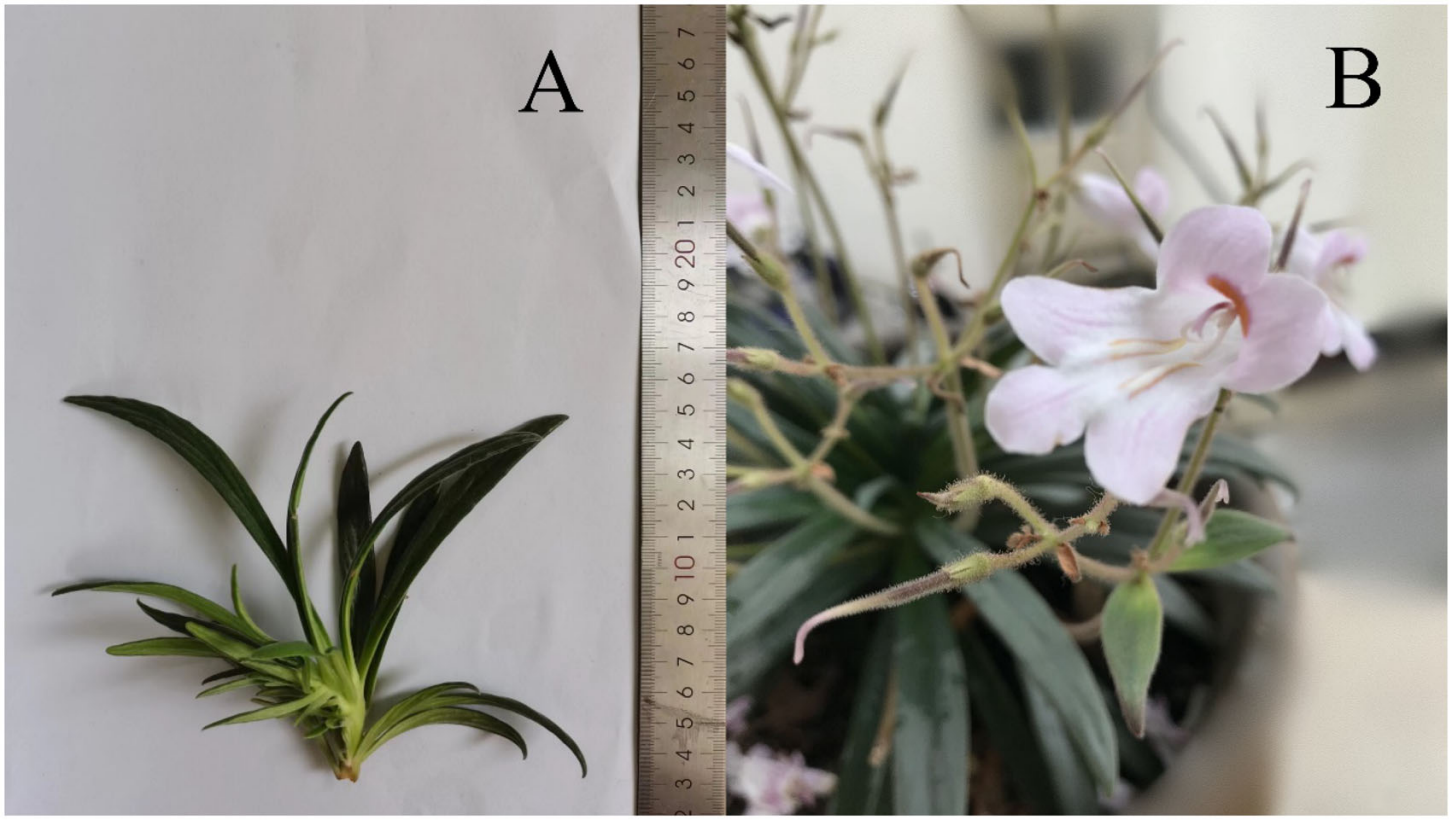
Reference image of *Primulina hedyotidea* taken by Haicheng Wen at the botanical garden of Guangxi University of Chinese Medicine.

We collected fresh leaves (Figure 1) in September 2021 from the campus of Guangxi University of Chinese Medicine (N22°48′14″; E 108°30′4″) and extracted the genomic DNA of *P. hedyotidea*. The collected specimen is stored in the Herbarium of Guangxi University of Chinese Medicine (voucher number 202109HJGC001) and can be accessed through the contact person Haicheng Wen (wenhaicheng2015@qq.com).

(A) Vegetative body. The plant is typically 12–18 cm tall with a long rhizome. The base leaf blade is oblong-lanceolate, 6.5–10 cm long, 0.9–2.4 cm wide, and has 3–4 lateral veins on each side. The flowers of *P. hedyotidea* are dish-cup-shaped and approximately 0.5 mm tall.

To obtain the original sequence data, we used the cetyltrimethylammonium bromide (CTAB) method (Roomi et al. 2013) to amplify the genomic DNA sequenced on an Illumina HiSeq4000 platform (Eo 2021). The second-generation sequencing data quality statistical software Cutadapt (version 1.9.1) was used to remove the linker and low-quality sequences from the pass filter data to obtain clean data for subsequent information analysis. Based on the optimized data, clean data were aligned to the reference genomic sequence using BWA (version 0.7.12) alignment software, and the aligned sequences were extracted. The segmented contig sequences were spliced using NOVOPlasty (version 2.7.2) software and further assembled into scaffold sequences with SSPACE (version 3.0) software. Finally, scaffold sequences with a low proportion of unknown N bases and long sequence lengths were obtained through processing with GapFiller (version 1-10) software. Predictive and noncoding ‘transfer RNA (tRNA)’ or ‘ribosomal RNA (rRNA)’ analysis of encoded genes was performed using the GeSeq (version 1.78) software and Rfam (version 12.0) database (Fan et al. 2020). CPGView (http://www.1kmpg.cn/cpgview) online tool was used to draw the *P. hedyotidea* genome circle and analyze difficult-to-annotate plastome genes (cis-splicing genes and trans-splicing genes) (Liu et al. 2023). The unique genes of *P. hedyotidea* chloroplast genome were annotated using CPGAVAS2 web service (Shi et al. 2019), then complete chloroplast genome sequence of the *P. hedyotidea* submitted to GenBank (Accession number: OM283823). To obtain the evolutionary history information of *P. hedyotidea*, we used MEGA X to construct a phylogenetic tree (Kumar et al. 2018). First, we downloaded the complete gene sequences of 14 species related to *P. hedyotidea* from GenBank and compared the chloroplast genome sequences with ClustalW sequences (Peng et al. 2022). Second, the maximum likelihood (ML) tree was produced using the Tamura-Nei substitution model (Tamura and Nei 1993) and the bootstrap method (1000 repetitions) for testing the phylogeny (Kumar et al. 2016).

## 3. Results

**Figure 2.**
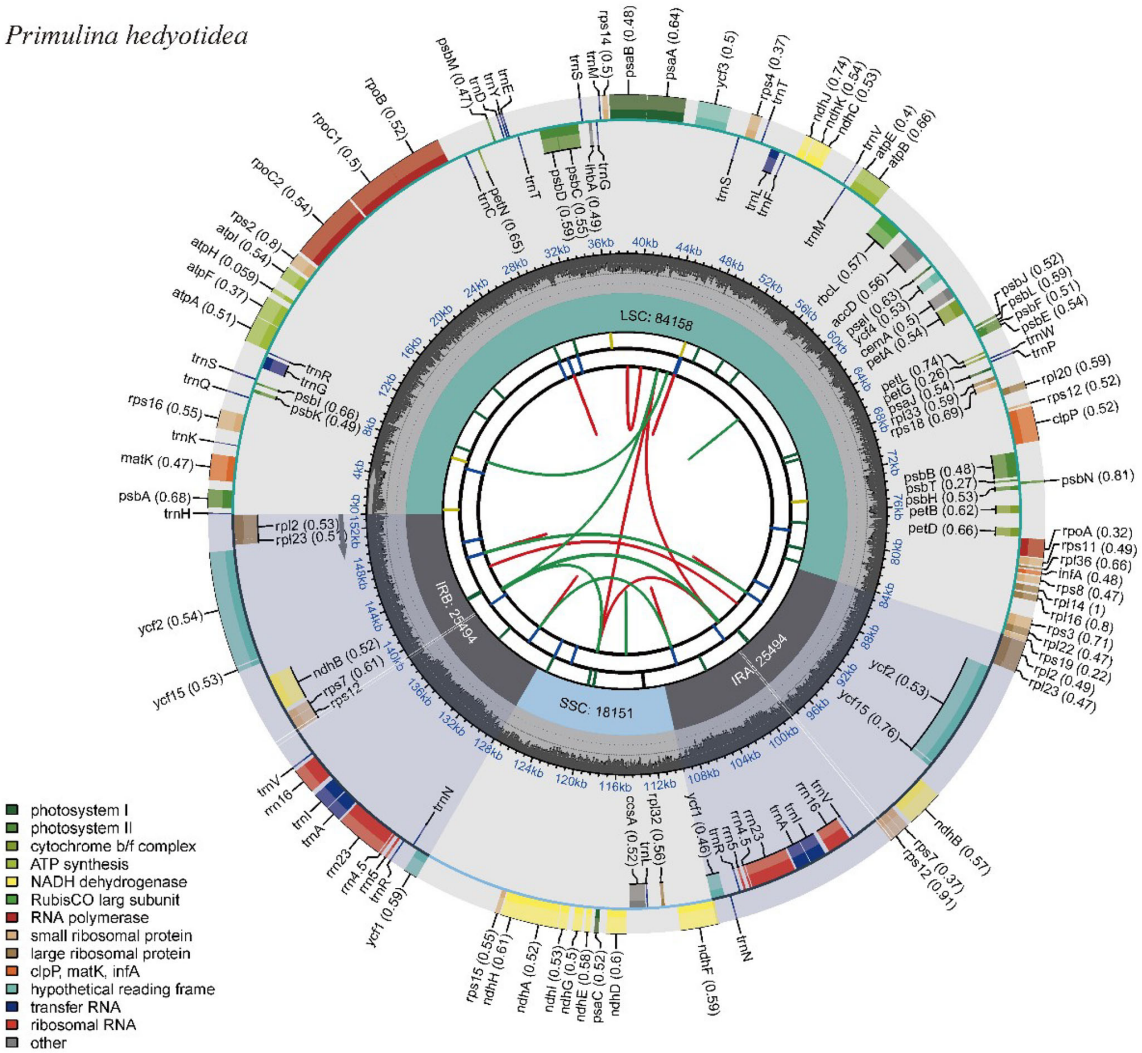
Chloroplast genome map of *Primulina hedyotidea.*

According to the assembly results, the length of the genome sequence was 153,297 bp, with an average depth of 542.90Χ (Supplementary Figure 1). The GC content was 37.62%, the inverted repeat (IR) region length was 25,494 bp, the large single copy (LSC) region was 84,158 bp and the small single copy (SSC) region was 18,151 bp (Table 1). In addition, the genome consisted of 80 protein-coding genes, 4 rRNA genes (*rrn16S*, *rrn23S*, *rrn4.5S*, *rrn5S*), and 28 tRNA genes, for a total of 112 genes (Figure 2). 80 protein-coding genes may be categorized into the following four groups based on their functions: A.44 photosynthesis-related genes, B.25 self-replication-related genes, C.7 other coding genes, and D.4 Unkown functional genes.

**Table 1.** Structure and composition of *P. hedyotidea* chloroplast genome.

Region	GC (%)	Length (bp)
Chloroplast genome	37.62	153,297
LSC	35.64	84,158
SSC	31.14	18,151
IRA	43.19	25,494
IRB	43.19	25,494

The circular chloroplast genome map displays 80 protein-coding genes, 28 tRNA genes, and 4 rRNA genes. Different categories of genes are labeled with distinct colors.

**Figure 3.**
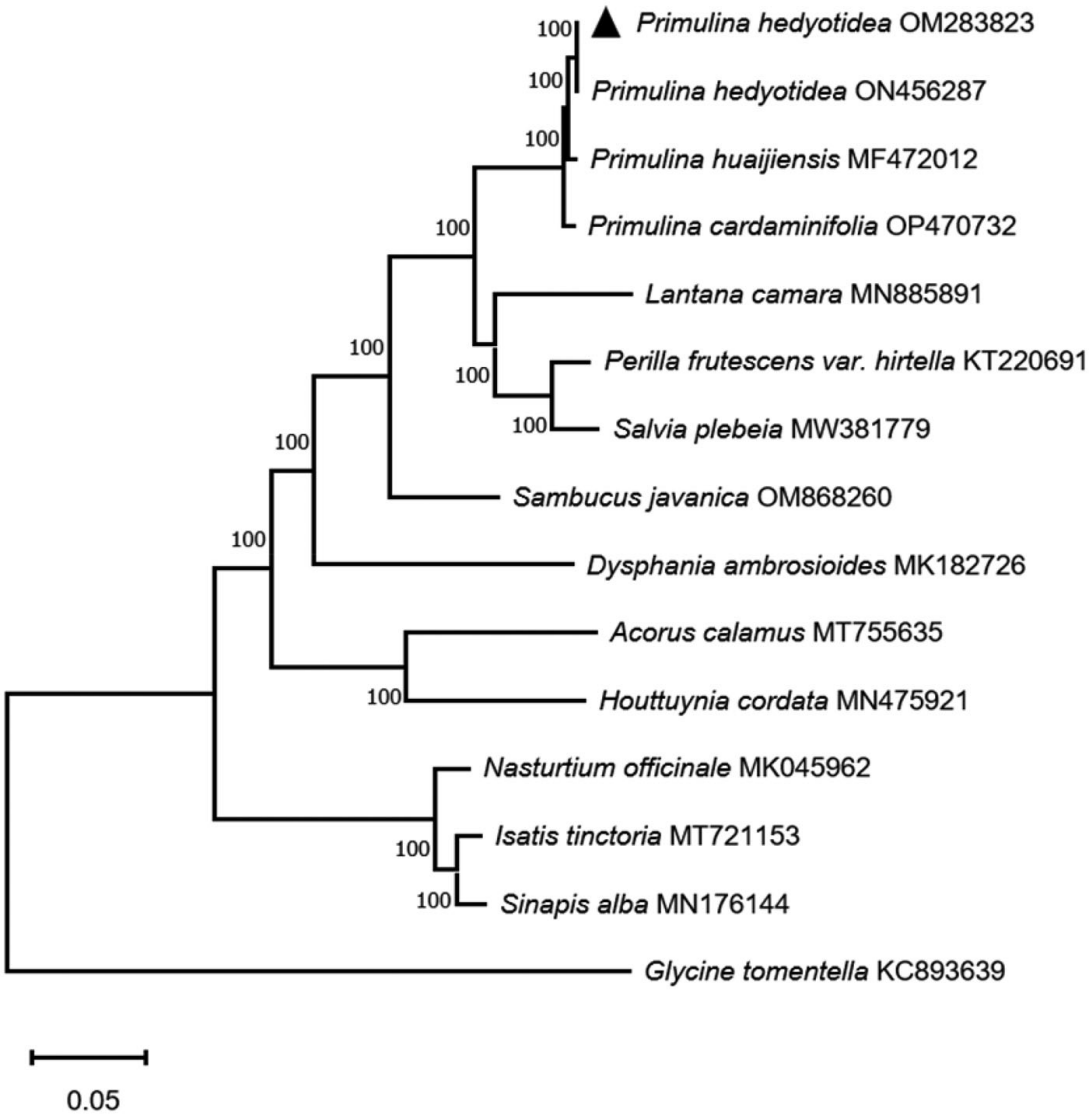
Maximum likelihood phylogenetic tree composed of the complete gene sequences of 14 related species obtained based on MEGA X analysis.

The medicinal plant *P. hedyotidea* and *Primulina huaijiensis* were found to be clustered together in the current experiment’s results (Figure 3), and species of the *Primulina* genus were found to be clustered together in the phylogenetic trees with a bootstrap value of 100, indicating that this genus was a confident monophyletic group. The cp genome information about *P. hedyotidea* provided by this study can help with species identification and phylogenetic analysis within *Primulina* and Gesneriaceae species. Due to the limitations of current technology, there are two separate sequence numbers for the same species since the sequence of a species may contain flaws that need ongoing updating and correction. Both the OM283823 and ON456287 complete genome sequences for *P. hedyotidea* were deposited in GenBank on November 27 and December 14, respectively. We compared OM283823 and ON456287 in terms of nucleotide composition and sequence accuracy. Supplementary Table 1 illustrates all eight single nucleotide polymorphisms. Regarding oligonucleotide differences between the two sequences, eight locations were identified (Supplementary Table 2). We found that the mapping reading, which is 292952, better supported OM283823 when we mapped the read to both genomes (Supplementary Table 3). In addition, we discovered the rps19 gene (Supplementary Table 4).

The following sequences were used: *Primulina hedyotidea* OM283823, *Primulina hedyotidea* ON456287, *Primulina huaijiensis* MF472012, *Primulina cardaminifolia* OP470732, *Lantana camara* MN885891, *Perilla frutescens var. hirtella* KT220691, *Salvia plebeia* MW381779, *Sambucus javanica* OM868260, *Dysphania ambrosioides* MK182726, *Acorus calamus* MT755635, *Houttuynia cordata* MN475921, *Nasturtium officinale* MK045962, *Isatis tinctoria* MT721153, *Sinapis alba* MN176144, *Glycine tomentella* KC893639.

## 4. Discussion and conclusion

For correction and annotation by multi-sequence alignment, cis-splicing in this study revealed that 14 genes (*rps16*, *atpF*, *rpoC1*, *rpl2*, *ndhB*, *ndhA*, *ndhB_copy2*, *rpl2_copy2*, *trnG-UCC*, *trnL-UAA*, *trnI-GAU*, *trnA-UGC*, *trnA-UGC_copy2*, and *trnI-GAU_copy2*) included one intron, four genes(*rps12*, *ycf3*, *rps12_copy2*, and *clpP*) contained two introns, one small-exon gene (*rps16*), and one trans-splicing gene (*rps12*), and the results are presented in Supplementary Figures 2 and 3. This study reports the chloroplast genome of *P. hedyotidea* for the first time and uses phylogenetic trees to analyze the relationships among related species. The phylogenetic tree indicates a close link between *Primulina huaijiensis* and *P. hedyotidea*, and this work adds new data to the phylogeny, species identification, conservation of species resources, and genetic investigations of the genus *Primulina*.

## Supplementary Material

Supplemental Material

## Data Availability

Genomic sequence data supporting the findings of this study are publicly available at the NCBI GenBank at https://www.nibi.nlm.nih.gov under accession number OM283823. The associated Bioproject, Bio-Sample, and SRA numbers are PRJNA801797, SAMN26021136, and SRR18059397, respectively.
