# Supplementary material for: Characteristics and phylogenetic analysis of the complete chloroplast genome of Primulina hedyotidea
Source: Mitochondrial DNA B Resour. 2023 Sep 23;8(9):1007–11. doi: 10.1080/23802359.2023.2238932 (PMC10519256; doi:10.1080/23802359.2023.2238932)
Supplement: Supplemental Material [file TMDN_A_2238932_SM7686.docx]

Figure captions

Supplementary Figure 1. Overall coverage depth of the chloroplast genome assembly of *Primulina hedyotidea*.

Supplementary Figure 2. Schematic map of the cis-splicing genes in the *P. hedyotidea* chloroplast genome.

Supplementary Figure 3. Schematic map of the trans-splicing gene rps12 in the *P. hedyotidea* chloroplast genome.


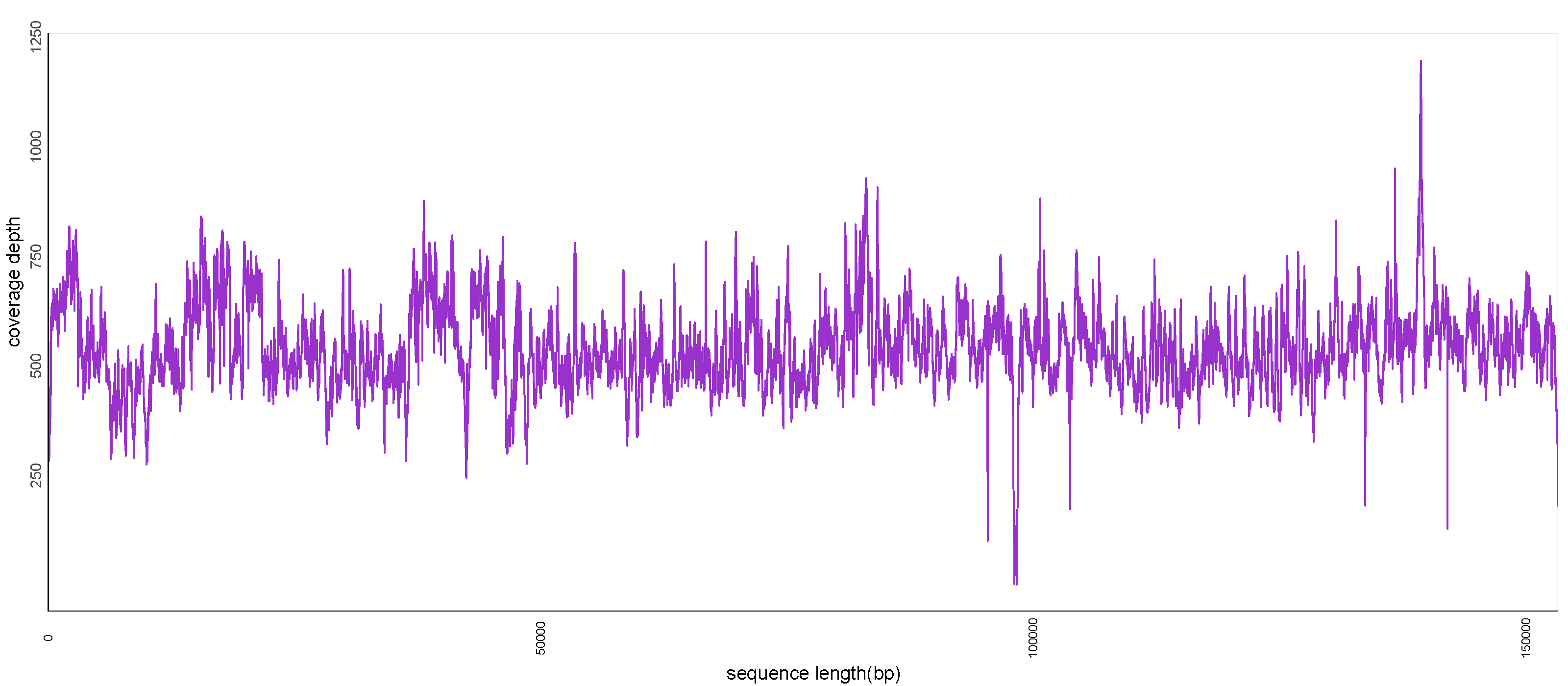


Supplementary Figure 1. Overall coverage depth of the chloroplast genome assembly of *Primulina hedyotidea*.


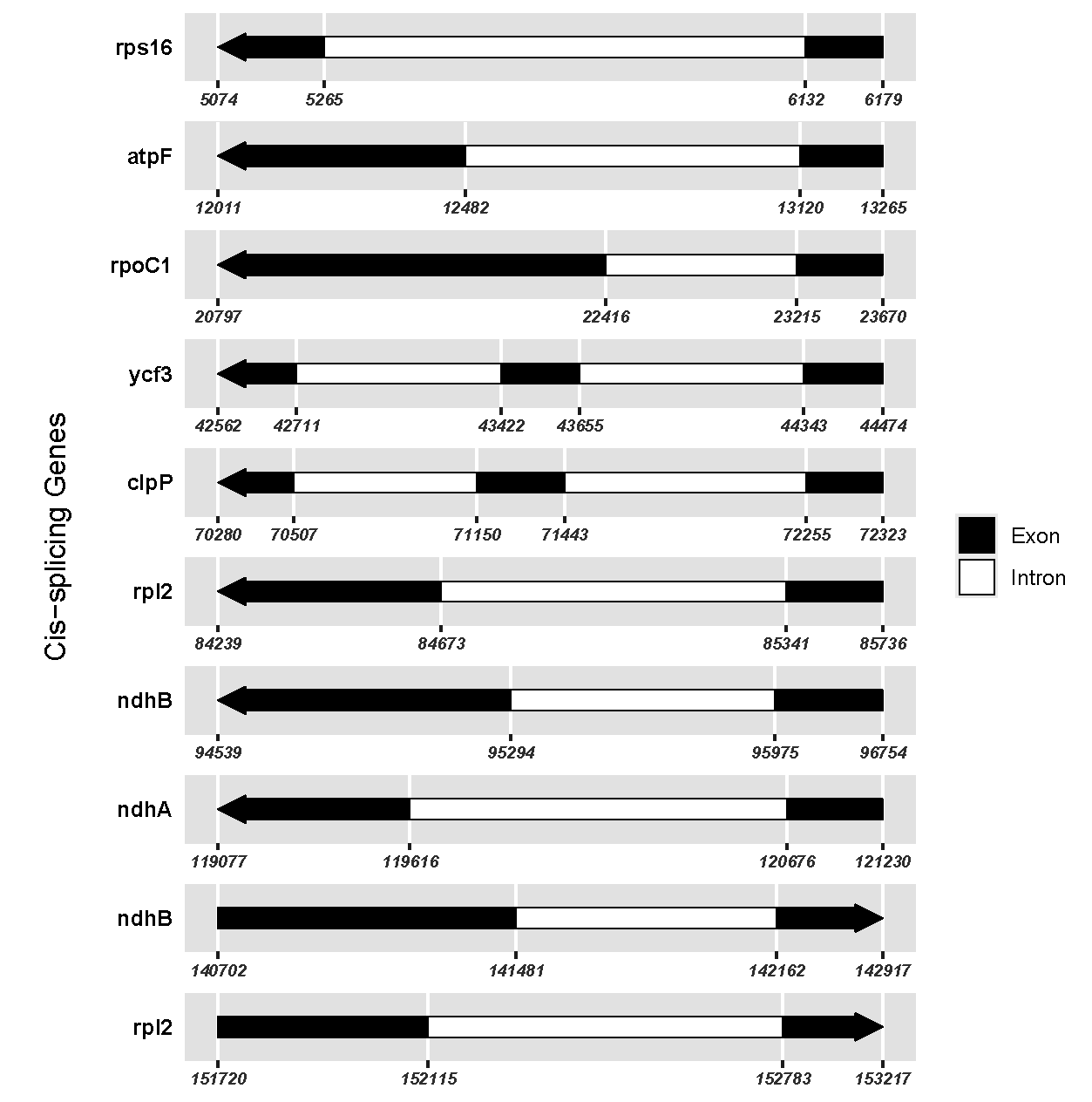


Supplementary Figure 2. Schematic map of the cis-splicing genes in the *P. hedyotidea* chloroplast genome.


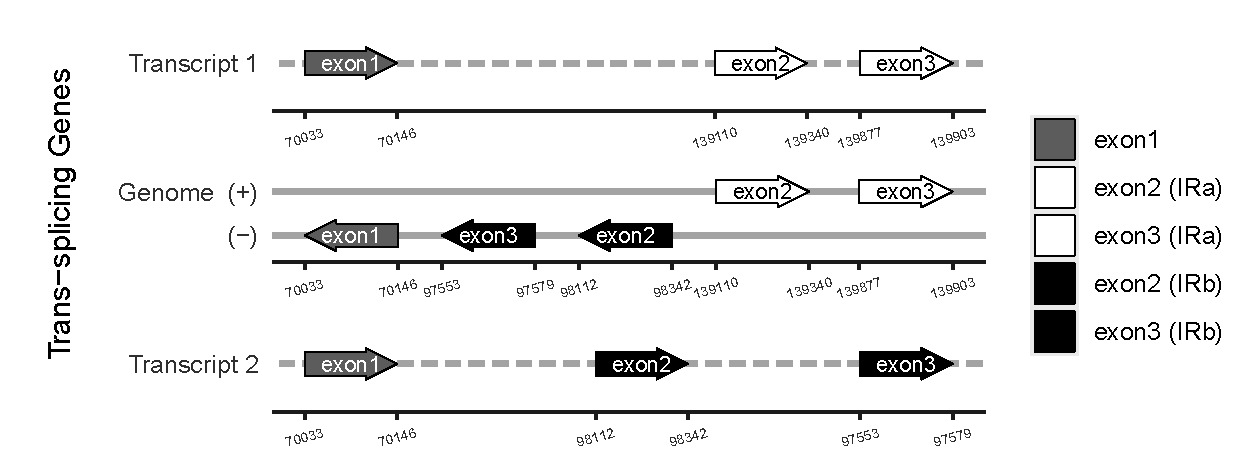


Supplementary Figure 3. Schematic map of the trans-splicing gene rps12 in the *P. hedyotidea* chloroplast genome.
